# The effect of addition of dexamethasone into normal saline irrigation solution on Prevention of Intraperitoneal Adhesion Post Laparotomy in wistar rats (Rattus norvegicus)

**DOI:** 10.1016/j.amsu.2020.08.053

**Published:** 2020-09-09

**Authors:** Dian Adi Syahputra, Asri Mashudy, Dasrul

**Affiliations:** a Pediatric Surgery Division, Department of Surgery, Faculty of Medicine, Syiah Kuala University/Dr. Zainoel Abidin Hospital, Banda Aceh, Aceh, Indonesia; b Resident of Surgery, Faculty of Medicine, Syiah Kuala University/Dr. Zainoel Abidin Hospital, Banda Aceh, Aceh, Indonesia; c Faculty of Veterinary Medicine, Syiah Kuala University, Banda Aceh, Aceh, Indonesia

**Keywords:** Intra peritoneal adhesion, Laparotomy, Dexamethasone, Normal saline

## Abstract

**Introduction:**

Intraperitoneal adhesion is a pathological condition of adhesion between the omentum, intestine and abdominal wall. The purpose of this study was to determine the effect of adding dexamethasone into normal saline fluid to prevent intra peritoneal adhesion.

**Materials and methods:**

This study used experimental post-test only control group design. There were four treatment groups using six white rats in each group: group I without administration of normal saline and dexamethasone, group II with administration of normal saline irrigation, group III by adding dexamethasone dose 0.2 mg/BW into 5 cc normal saline, and group IV by adding 0.5 mg/BW into 5 cc normal saline. Laparotomy was performed to all samples followed by excoriation and abrasion in cecum and terminal ileum using gauze. Intra peritoneal adhesion assessment was carried out on the 10th day post laparotomy. Macroscopic and microscopic assessments were performed to evaluate formation of collagen, fibrin and the spread of inflammatory cells of each group.

**Results:**

From macroscopic observations, it was found that the average adhesion that occurred was 3.333; 2.333; 0.666; 0.333 by using statistical calculations with one-way ANOVA with *P* value of 0.000. Post-Hoc analysis showed that the administration of dexamethasone 0.5 mg/BW is proven to minimize the occurrence of adhesion between the 3 groups. Microscopic assessment of the formation of collagen, fibrin and the spread of inflammatory cells by one-way ANOVA produced *P* 0.000 in all three parameters, but the dose of dexamethasone administration between 0.2 mg/BW and 0.5 kg/BW did not prove significant in either group.

**Conclusion:**

There is evidence that the addition of dexamethasone to normal saline as an irrigation liquid during laparotomy can reduce the occurrence of adhesion. However, the dose difference was not proven to be better in this study. Further studies are suggested to use more experimental animals.

## Highlights

• Administration of dexamethasone was proven to reduce the amount of the three parameters.
• Addition of dexamethasone to normal saline as an irrigation liquid during laparotomy can reduce the occurrence of adhesion.
• Administration of normal saline can reduce inflammatory cells in the intraperitoneal adhesion tissue.

## 1 Introduction

Intraperitoneal adhesion is a pathological condition of adhesion between omentum, intestines and abdominal wall. Adhesion could be thin connective tissue, such as fibrosis, that contains blood vessels and neural tissues, or a direct contact between two surface organs [1]. Generally, adhesion is caused by surgical procedures in abdominal and peritoneal cavity. Approximately 1/3 patients undergoing abdominal or an open pelvic surgery are two-time more likely to develop adhesion in the next 10 years. Post-surgery adhesion leads to an increase in inpatient episodes, duration of operation time and cost due to impaired bowel obstruction [1,2]. In North America, 300.000 hospitals treated 850.000 patients with adhesion small bowel obstruction (ASBO) which spent more than USD 1.3 billion in medical costs and caused 2000 deaths annually [3].

In abdominal surgery, peritoneal injury during surgery, foreign bodies, infections and or irritation will initiate inflammation resulting in fibrinous exudate and fibrin formation along with inflammatory cells, macrophages, lymphocytes, platelets and degradation products (interleukin and interferon). These components play a role in the formation and degradation of post-surgical extracellular matrix. During surgery, interruption of fibrinolysis could trigger imbalance between fibrin deposition and degradation. In normal condition, degradation of fibrin makes peritoneal healing without adhesion. Conversely, the undegraded fibrin would trigger fibroblasts and capillaries to form intraperitoneal adhesion [4,5].

Before the closure of peritoneum at the end of laparotomy operation, irrigation of abdominal cavity is commonly done, generally by using normal saline about 500–1.000 mL or as needed. The objective is to remove blood remnants and tissues to avoid complications of adhesion after surgery [6,7]. Corticosteroids are reportedly able to reduce inflammatory responses by reducing vascular permeability and releas of cytokines as well as the chemotactic factors, which play a role in the formation of adhesions. Dexamethasone has a significant steroid effect in a functional cytokine induced intercellular-1 adhesion molecules (ICAM-1), which is functionally associated with the ability to suppress the overall transendothelial neutrophil. ICAM is a possible molecular target for the anti-inflammatory action of dexamethasone [8]. Inhibition of ICAM-1 regulation may or at least partially mediate the potential antimigration action demonstrated by this anti-inflammatory drug in suppressing adhesion.

## 2 Methods

Ethical approval has been approved by the local Veterinary Ethics Committee. This work is has been reported in line with the ARRIVE statement [9]. Using experimental design, a post-test only control group was chosen by involving white male rats (Rattus norvegicus) as the research subjects with these characteristics: 3–4 months old and weighed 200–300 g. These rats were then divided into 4 groups: group without administration of normal saline and dexamethasone (group 1), group with administration of normal saline irrigation (group 2), group with administration of normal salin and additional 0.2 mg/BW dexamethasone in 5 cc normal saline (group 3), and group with administration of normal salin and additional 0.5 mg/BW dexamethasone in 5 cc normal saline (group 4). The determination of the number of samples for each group is based on Federer's formula, resulting in minimum of six sample per group.

### 2.1 The preparation of experimental animals

A total of 24 white rats (Rattus Novergicus) were prepared for the experiment. Before the treatment, some steps were carried out as follows: the rats were treated the same way, weighed and adapted for 1 week, homogenization was carried out in the cage, the temperature in the cage was set at room temperature, and the rats were fed 20 g of pellets and given water ad libitum every day. There was no exclusion. No randomization was done.

### 2.2 The dexamethasone dose

In group 3, the dose given was 0.2 mg/kgBW in 5 mL NaCl 0.9%. In humans weighing 70 kg, the dose is 70 kg × 0.2 mg = 14 mg. Human conversion dose (0.018) with a weight of 70 kg is 14 mg x 0.018 = 0.252 mg. So, for group 3, each rat was given 0.252 mg dexametahose in 5 mL. In group 4, the dose given was 0.5 mg/kgbb. In humans weighing 70 kg, the dose is 70 kg × 0.5 mg = 35 mg. Human conversion dose (0.018) with a weight of 70 kg is 35 mg x 0.018 = 0.63 mg. So, for group 4, each rat was given 0.63 mg/5 mL dexametahose in 5 mL.

### 2.3 The laparotomy surgical procedure

**Fig. 1:**
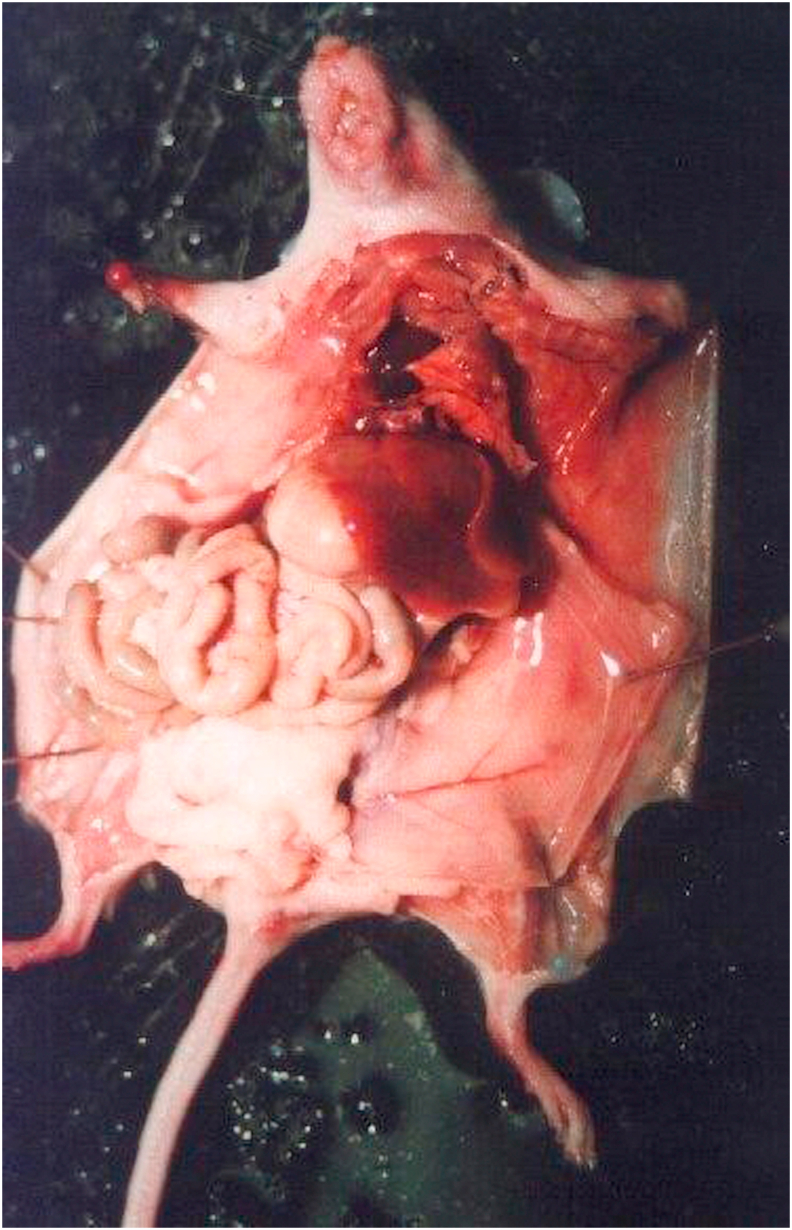
Laparotomy procedure.

The rats are subjected to anesthesia with ketamine 30 mg/kg BW through the femoral and they breath spontaneously during the procedure. The abdominal area was shaved, then the asepsis technique was done with povidone-iodine 10% and alcohol 70%. Laparotomy was performed (Fig. 1) with a 4 cm midline abdomen incision. Exploration was carried out to find the ileocaecal junction (ICJ). Cecum and terminal ileum (1 cm from ICJ) were excoriated and abrased using gauze to trigger bleeding spots of at least one to two cm. The same procedure was also performed on the same side of the abdominal wall with the position of the cecum and terminal ileum of 1–2 cm until the bleeding spots occurred. Surgical abdominal wound suturing was immediately performed in group 1 with simple interrupted sutures using silk 5.0 and covered with gauze. Group 2 was irrigated with normal saline liquid as much as 5 cc, left to stand for 10 min and then dried using sterile gauze. In the treatment groups 3 and 4, there was an additional dexamethasone as much as 0.2 mg/kg BW and 0.5 mg/kg BW in 5 mL normal saline then continued with irrigation into the intraabdominal slowly up to the location of abrasion and abdominal wall. The irrigation liquid was left for 10 min then drained with sterile gauze. Laparotomy incision wounds are primary sutured with simple interrupted sutures using silk 5.0. The mice were given prophylactic antibiotics of intra muscular ceftriaxone 50 mg/kg BW (human dose 70 kg × 50 mg/kg BW = 3500 mg) so that the dose given to the mice weighing 200–300 g is 3500 mg x 0.018 = 63 mg.

### 2.4 The macroscopic intraperitoneal adhesion assessment

Assessment of intraperitoneal adhesion was carried out on the 10th day after the administration of intraperitoneal fluid surgery and irrigation. Termination of euthanasia was done with lethal dose of chloroform by inhalation in all experimental animals. Contralateral midline incision from the previous incision on the abdomen was widened to the cranial and caudal to make access to the abdominal cavity to find the location of intraperitoneal adhesion. Macroscopic adhesion assessment using Nair criteria (Table 1) [10].

**Table 1:** Nair criteria.

Score	Description
1	No adhesion
2	There is 1 adhesion between the viscera/abdomen wall
3	There is 2 adhesion between the viscera/abdomen wall
4	There are >2 visceral/abdominal wall adhesions or the entire intestine without mass in the abdominal wall
5	There is a broad visceral abdominal adhesion

### 2.5 The microscopic intraperitoneal adhesion assessment

After macroscopic assessment, microscopic assessment was performed. Intraabdominal tissue undergoing adhesion was taken for histological examination. We took 2 cm of the tissue and then fixed it with 10% formaldehyde buffer, and then the paraffin block was made to be stained with HE staining. The sample was dried and examined with a 100× magnification light microscope. Evaluation was carried out by an anatomic pathologist to assess the condition of fibrin, collagen and inflammatory cells in intraperitoneal adhesion.

Fibrin in intraperitoneal adhesion tissue was assessed using a 100× magnification microscope with DeRoy van Zuidewijn et al. (1992) criteria (Table 2) [11]. The collagen arrangement in intraperitoneal adhesion tissue was assessed using a 100× magnification microscope with Calvi et al. criteria (Table 3) [12]. The microscopic distribution of the inflammatory response in intraperitoneal adhesion tissue was assessed using a 100× magnification microscope with Woods et al. (1984) criteria (Table 4) [13].

**Table 2:** DeRoy van Zuidewijn et al. criteria.

Score	Description
0	No visible accumulation of fibrin fiber
1	Mild fibrin accumulation
2	Medium fibrin accumulation
3	Complete adhesion with many fibrin findings

**Table 3:** Calvi et al. criteria.

Score	Description
0	There is no collagen composition
1	Minimal collagen arrangement
2	Medium collagen arrangement
3	A lot of composition of collagen

**Table 4:** Woods et al. criteria.

Score	Description
0	Inflammatory cellular infiltration with little evidence of fibroplasias
1	Lack of inflammatory cells and evidence of early fibroplasia
2	There is a decrease in inflammatory cells in the presence of immature fibroplasia
3	The presence of active and mature fibrocytes

Macroscopic and microscopic adhesion data which were obtained in all treatment groups were tested for normality and homogenesity tests. Data that were normally distributed were then analyzed with ANOVA, values were considered significant (*P* < 0.05) followed by least significant difference (LSD) test to see differences between treatments. Statistical analysis was performed with the help of Software Statistical Product and Solutions (SPSS) Version 17.0 for Windows.

## 3 Results

### 3.1 Macroscopic profile intraperitoneal adhesion

**Fig. 2:**
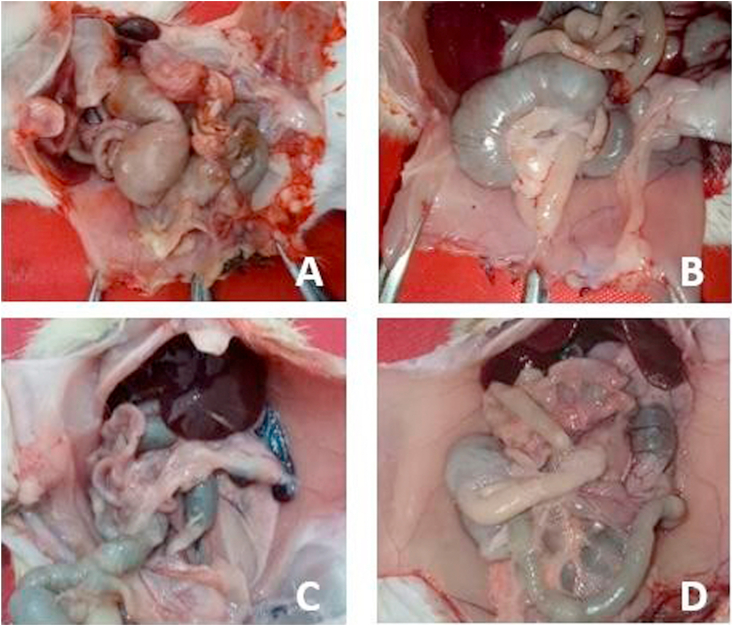
Macroscopic degree of intraperitoneal adhesion.

No Wistar rats died during the study period until termination. The results of observations of the macroscopic picture of the intraperitoneal adhesion degree after Wistar rat laparotomy in each treatment could be seen in Fig. 2. The average degree of intraperitoneal adhesion after laparotomy in each treatment could be seen in Table 5.

**Table 5:** The average degree of intraperitoneal adhesion after laparotomy procedure.

Sample	Treatement Groups				*one way* ANOVA
	Group 1	Group 2	Group 3	Group 4	
1	4	3	0	0	*P* = 0,000
2	2	2	0	0	
3	3	2	0	0	
4	4	3	1	1	
5	4	3	1	0	
6	3	1	2	1	
Mean	3.333	2.333	0.666	0.333	
SD	0.816	0.816	0.816	0.516	

### 3.2 Microscopic profile of intraperitoneal adhesion

**Fig. 3:**
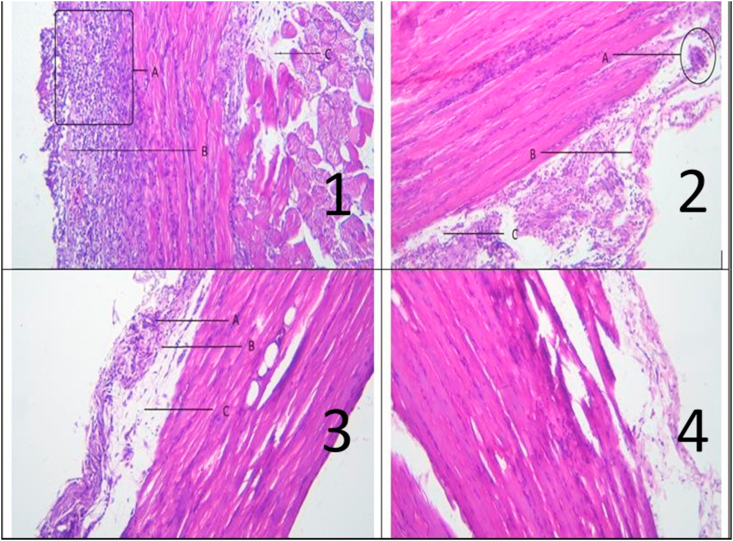
Histology picture of intraperitoneal adhesion.

The results of observation to the microscopic picture of intraperitoneal adhesion tissue which was observed based on the amount of fibrin, collagen composition and inflammatory cells could be seen in Fig. 3.

From the picture above, it could be seen that the amount of fibrin, collagen and inflammatory cells increases rapidly in the negative control group, then decreases successively and decreases in the positive control group and the treatment group.

The average amount of fibrin, collagen composition and the distribution of inflammatory cells in the intraperitoneal adhesion tissue after laparotomy in each treatment could be seen in Table 6, Table 7, Table 8.

**Table 6:** The average amount of fibrin in the intraperitoneal adhesion tissue after laparotomy.

Sample	Treatment Groups				*one way* ANOVA
	Group 1	Group 2	Group 3	Group 4	
1	2	1	1	0	*P* = 0.000
2	3	2	1	0	
3	2	1	0	1	
4	3	1	0	0	
5	1	2	1	0	
6	2	1	0	1	
Mean	2.166	1.333	0.500	0.333

**Table 7:** The average amount of collagen composition in the intraperitoneal adhesion tissue after laparotomy.

Sample	Group Treatment				*one way* ANOVA
	Group 1	Group 2	Group 3	Group 4	
1	2	1	0	0	*P* = 0.000
2	2	1	0	1	
3	2	2	1	1	
4	3	1	1	0	
5	1	1	0	0	
6	3	1	1	0	
Mean	2.166	1.166	0.500	0.333

**Table 8:** The average distribution of inflammatory cells in the intraperitoneal adhesion tissue after laparotomy.

Sample	Group Treatment				*one way* ANOVA
	Group 1	Group 2	Group 3	Group 4	
1	3	2	1	2	*P* = 0.000
2	3	3	2	1	
3	4	3	2	1	
4	4	3	3	2	
5	4	2	1	2	
6	3	2	2	1	
Mean	3.500	2.500	1.833	1.500	

From the three tables above, it was found that the average amount of fibrin, collagen composition and distribution of inflammatory cells in the intraperitoneal adhesion tissue in group 4 was better than the other groups.

Significant differences between groups in assessing the amount of fibrin, collagen composition and distribution of inflammatory cells in microscopic intraperitoneal adhesion tissue could be further determined by post hoc analysis (Table 9).

**Table 9:** Post Hoc analysis between groups in assessing the amount of fibrin, collagen composition and distribution of inflammatory cells.

Group	Comparison Groups	P-value			
		Adhesion	Fibrin	Collagen	Inflammatory cells
Group 1	Group 2	0.032	0.024	0.006	0.010
	Group 3	0.000	0.000	0.000	0.000
	Group 4	0.000	0.000	0.000	0.000
Group 2	Group 1	0.032	0.024	0.006	0.010
	Group 3	0.001	0.024	0.056	0.071
	Group 4	0.000	0.008	0.020	0.010
Group 3	Group 1	0.000	0.000	0.000	0.000
	Group 2	0.001	0.024	0.056	0.071
	Group 4	0.452	0.631	0.618	0.352
Group 4	Group 1	0.000	0.000	0.000	0.000
	Group 2	0.000	0.008	0.020	0.010
	Group 3	0.452	0.631	0.618	0.352

## 4 Discussion

Adhesion to negative control had the most severe degree, followed by the positive control group using normal saline and the dexamethasone treatment group, as seen in Fig. 2. In this study, statistical tests were performed using one way ANOVA method which aims to find differences in the degree of adhesion in the tested animals. The results obtained were *P* = 0.00 (*P* < 0.05) which indicates that there is a significant difference in the degree of macroscopic intraperitoneal adhesion in wistar rats. There was a significant difference in group 4 for fibrin, collagen composition and distribution of inflammatory cells compared to the other three groups. This explains that the administration of dexamethasone to a normal saline fluid as peritoneal irrigation fluid is proven to minimize the occurrence of intraperitoneal adhesion.

Based on the post hoc analysis with BNT test, the mean of intraperitoneal adhesion, the amount of fibrin, collagen composition and the distribution of inflammatory cells in the intraperitoneal adhesion tissue of white rats in group 4 were significantly lower compared to groups 1 and 2 with *P*-values <0.05. Significant difference was also found in group 3 compared to group 1 in, the mean degree of intraperitoneal adhesion, the amount of fibrin, collagen composition and the distribution of inflammatory cells in the intraperitoneal adhesion tissue. No significant difference was found in the dexamethasone addition treatment to normal saline fluid with different doses group in post hoc analysis.

Based on post hoc analysis, group 2 was significantly different to group 1 (*P* = 0.032). This shows that the administration of NaCl 0.9% could reduce intraperitoneal adhesion. Normal saline could get rid of the remaining blood, the remaining pieces of tissue and other fluids to avoid complications in the form of postoperative adhesion [6,7,14]. This study shows that the normal use of saline decreases fibrinolytic capacity. The decrease of fibrinolytic capacity will reduce the incidence of intraperitoneal adhesion after laparotomy. The results of this study are in accordance with the research conducted by Winckiewicz et al. (2007).^7^ In their study, normal saline could be used to reduce the risk of intraperitoneal adhesion by inducing mesothelial cell deterioration and reducing fibrinolytic activity [6,15].

Group 3 and group 4 had significant differences with group 2 with *P* values = 0.001 and 0.000 (*P* < 0.05) respectively, which shows that the treatment group using normal saline with additional dexamethasone 0.2 mg/kg BW and dexamethasone 0.5 mg/kg BW has an influence on decreasing intraperitoneal adhesion compared to the positive control group. This is consistent with the research conducted by Wheller et al. (1997), who showed that using postoperative dexamethasone could significantly reduce inflammatory mediators by decreasing the expression of intercellular-1 adhesion molecules (ICAM-1) in three different endothelial cell lines that were modulated by pro cytokines pro-inflammation, such as interleukin-1β, tumour necrosis factor-alpha (TNF-alpha) and dexamethasone glucocorticoid hormones [8]. This was also supported by the research conducted by Wang et al.(2003), who conducted research using AMD combination therapy (allantoin, metronidazole and dexamethasone) in canine intraabdominal adhesion and found that the combination of AMD was an effective way to prevent intra-abdominal adhesion [16].

Formation of fibrin fibres was also significantly lower in group 2 compared to group 1 with *P* = 0.024 (*P* < 0.05). This shows that the administration of normal saline can reduce the amount of fibrin in the intraperitoneal adhesion tissue. The results of this study are following the research conducted by Winckiewicz et al.(2007), who found that normal saline fluid could be used to reduce the risk of intraperitoneal adhesion by inducing mesothelial cell deterioration and decreasing fibrinolytic activity [7]. Groups 3 and 4 also had significant differences in group 2 with *P* values = 0.024 and 0.008 (*P* < 0.05) respectively. This is in line with the research of Yudaniayanti et al. (2012), who used a mixture of ampicillin, dextran 40 and dexamethasone on intraperitoneal adhesion scores. It was seen that the adhesion score decreased in the dexamethasone group as well as a combination of antibiotics and dextran compared to controls [17]. Ionescu et al. (2018) also investigated the effects of dexamethasone administered before laparoscopic action to significantly reduce ICAM-1 levels in blood plasma [18]. The results of this study were also in line with the results of the study of Wheller et al.(1997), in which there is a significant effect of steroids on the regulation of ICAM-1 that is functionally triggered by cytokines related to its ability to suppress in vitro neutrophil transendothelial which overall shows that ICAM-1 is a possible molecular target for anti-action -inflammation carried out by dexamethasone [8]. Fibrinolytic agents can cause bleeding complications, but recombinant forms of t-PA when applied topically, reduce adhesion in animal models without increasing complications. This approach is promising in preventing postoperative adhesions. The effectiveness of rt-PA obtained by recombinant DNA techniques has been studied in the prevention of primary adhesion and recurrence. In this experimental model, fibrinolytic activity is reduced by the presence of thermal or mechanical trauma, ischemia, and inflammatory factors that are known to cause adhesion formation [6,10].

In collagen formation parameter, group 2 was significantly lower compared to group 1 with *P* = 0.006 (*P* < 0.05). This shows that the administration of normal saline can reduce collagen formation in the intraperitoneal adhesion tissue. According to the theory put forward by Buåureanu, the initial phase of bio cellular healing process in peritoneum is done by stimulating coagulation cascade system which will produce fibrin strands in peritoneal cavity in the presence of fibrin, which stimulates the formation of adhesion through increased fibroblast activity stimulated by growth factors i.e. PDGF and TGF beta, fibroblasts; and mesothelial cells will deposit collagen fibres to form fibrous adhesion [1]. At present, there are no studies that report the effect of normal saline on collagen formation in intraperitoneal adhesion tissue [7]. Group 3 did not have significantly lower formation from group 2 (*P* = 0.056), but group 4 had significantly lower formation compared to group 2 (*P* = 0.020). Robert et al. (1965) who conducted a study using a combination of promethazine and dexamethasone showed significantly lower rate of adhesion by suppressing fibrin production so that it also reduces collagen formation in adhesion tissue [13].

Group 1 had higher inflammatory cells compared to group 2 (*P* = 0.010). This shows that administration of normal saline can reduce inflammatory cells in the intraperitoneal adhesion tissue. The results of this study are consistent with the research of Aydin et al. (2018) who researched the effect of Argan oil and normal saline on the degree of intraperitoneal adhesion, and found out that normal saline administration and Argan oil can reduce the degree of adhesion in intraperitoneal tissue macroscopically and also found a significant decrease in Giant cell, neutrophils, lymphocytes, plasmocytes which are inflammatory cells [19]. Group 3 did not differ significantly from group 2 (P = 0.071), while group 4 had lower inflammatory cells compared to group 2 (P = 0.010). This showed that steroid use had a significant effect on inflammatory cells in intraperitoneal adhesion tissue. Ionescu et al. (2018) in a study using dexamethasone at a dose of 4 mg which is given before laparoscopic cholecystectomy surgery showed a decrease in the inflammatory response. This effect was mediated by increasing interleukin anti-inflammatory plasma levels and decreasing interleukin proinflammation [18]. This is in line with the research conducted by us using dexamethasone mixed with normal saline as a postoperative irrigation fluid. Group 3 and group 4 did not have significant differences (P = 0.352). This shows dose difference did not give lower inflammatory response in the tested animals. In Kirdak et al.‘s study (2008) who assessed the effectiveness of methylprednisolone doses to prevent intraabdominal adhesions with a dose of 16 mg/kg BW and 10 mg/kg BW which is given into peritoneal and topical has showed no significant differences between groups of tested animals [20]. This is consistent with the results of the present study that there was no difference in the effects of dexamethasone administration at a dose of 0.2 mg/kg BW and 0.5 mg/kg BW as a prevention of postoperative intraperitoneal adhesion.

Our study used calculated sample size to make sure the number of subjects needed for the study. All the subjects were managed carefully and there were no loss of data in the process. One of the limitations of the study was there was no randomization, concealment, and blinding in the experimentation process. This could lead to high risk of bias towards the result of the study.

## 5 Conclusions

There is evidence that the addition of dexamethasone to normal saline as an irrigation liquid during laparotomy can reduce the occurrence of adhesion. However, the dose difference was not proven to be better in this study.

## Provenance and peer review

Not commissioned, externally peer reviewed.

## Declaration of competing interest

The authors declare that there is no conflict of interest regarding publication of this paper.

## Annals of medicine and surgery

The following information is required for submission. Please note that failure to respond to these questions/statements will mean your submission will be returned. If you have nothing to declare in any of these categories then this should be stated.
